# Orosensory contributions to dysphagia: a link between perception of sweet and sour taste and pharyngeal delay time

**DOI:** 10.14814/phy2.12752

**Published:** 2016-06-14

**Authors:** Barbara R. Pauloski, Sazzad M. Nasir

**Affiliations:** ^1^ Department of Communication Sciences and DisordersUniversity of Wisconsin‐MilwaukeeWisconsin; ^2^ Department of Communication Sciences and DisordersNorthwestern UniversityIllinois

**Keywords:** Citric acid, deglutition, deglutition disorders, sucrose, taste perception

## Abstract

Pharyngeal delay is a significant swallowing disorder often resulting in aspiration. It is suspected that pharyngeal delay originates from sensory impairment, but a direct demonstration of a link between oral sensation and pharyngeal delay is lacking. In this study involving six patients with complaints of dysphagia, taste sensation of the oral tongue was measured and subsequently related to swallowing kinematics. It was found that a response bias for sour taste was significantly correlated with pharyngeal delay time on paste, highlighting oral sensory contributions to swallow motor dysfunctions. Investigating the precise nature of such a link between oral sensation and dysphagia would constitute a basis for understanding the disorder. The results of this study highlight oral sensory contributions to pharyngeal swallow events and provide impetus to examine this link in larger samples of dysphagic patients.

## Introduction

Pharyngeal swallow delay is one of the most common and significant oropharyngeal swallowing disorders, presenting in high frequency in patients with dysphagia resulting from neurologic disorders such as stroke and Parkinson's disease and those with treatment for head and neck cancers (Logemann and Bytell 1979; Veis and Logemann 1985; Logemann et al. 1993a,b; Pauloski et al. 1993; Bisch et al. 1994; Abraham and Yun 2002; Warabi et al. 2008; Terré and Mearin 2012). Pharyngeal swallow delay frequently results in aspiration of thin liquids and potentially pneumonia (Morton et al. 2002; Kim and McCulloogh 2007; Warabi et al. 2008; Bingjie et al. 2010; Kang et al. 2011; Terré and Mearin 2012). There is evidence that delay in triggering the pharyngeal swallow is related to a sensory disorder in which the cortex and brainstem take longer to recognize sensory input regarding the need to swallow and to trigger the pharyngeal stage of swallow (Power et al. 2007; Warabi et al. 2008). Stroke patients with dysphagia have been shown to demonstrate significant sensory deficits in the laryngopharynx that likely contribute to the development of aspiration (Aviv et al. 1996, 1997; Dziewas et al. 2004).

Sensory stimulation is essential for an efficient and organized oropharyngeal swallow. The reflexive pharyngeal swallow and laryngeal adduction may be activated by stimulation of the internal branch of the superior laryngeal nerve of the vagus (CN X) and the pharyngeal branch of the glossopharyngeal nerve (CN IX) in both animals and humans (Doty 1951; Doty and Bosma 1956; Sinclair 1971; Ludlow et al. 1992; Barkmeier et al. 2000; Kitagawa et al. 2002). Sensory fibers from CN X and CN IX travel to the medulla where they synapse in the nucleus tractus solitarius (NTS). The NTS is the primary location of the dorsal swallow group, part of the presumed swallow central pattern generator (CPG) that theoretically is responsible for the timing and coordination of the pharyngeal swallow (Miller 1999a). The swallow CPG involves several motor and sensory nuclei and interneurons in the medulla. The dorsal swallow group, located within the NTS and the adjacent reticular formation, contains neurons involved in triggering, shaping, and timing sequential swallowing patterns. In addition to peripheral inputs from the cranial nerves, the dorsal swallow group also receives supramedullary inputs from cortical and subcortical structures. Via interneuronal connections within the CPG, the neurons in the dorsal swallow group activate those in the ventral swallow group located in the ventrolateral medulla. The ventral swallow group contains switching neurons that distribute swallowing drive to pools of motorneurons and preganglionic neurons in the brainstem (Jean and Dallaporta 2013). The swallow CPG therefore may be activated by either peripheral or cortical inputs.

Several studies have demonstrated that controlled air pulse stimulation to the soft palate or anterior faucial pillars will activate both motor and sensory cortical and subcortical regions related to active swallowing (Yoshida et al. 2006; Lowell et al. 2008); however, sensation from these structures is mediated by the glossopharyngeal nerve (CN IX), stimulation of which is already known to be able to elicit a swallow. In addition to afferent input from CN X and CN IX, the NTS receives sensory input from the oral tongue via fibers of the chorda tympani branch of the facial nerve (CN VII) for taste on the anterior two‐thirds of the tongue. It has not been demonstrated that sensory stimulation of the oral tongue exclusively can evoke a pharyngeal swallow response.

Previous research on both healthy control subjects (Palmer et al. 2005; Pelletier and Steele 2014) and patients who suffered central or peripheral neurologic impairment (Logemann et al. 1995; Pelletier and Lawless 2003; Cola et al. 2012; Lee et al. 2012; Pauloski et al. 2013) demonstrated changes in swallow physiology when given a sour stimulus. Specifically, with a sour bolus, subjects demonstrated higher anterior palate pressures and stronger submental electromyographic activity (Palmer et al. 2005; Pelletier and Steele 2014), shorter oral transit times (OTTs) (Lee et al. 2012), faster onset of the oral stage and the triggering of the pharyngeal swallow (Logemann et al. 1995; Lee et al. 2012), shorter pharyngeal transit times (PTTs) for sour boluses combined with cold (Cola et al. 2012), fewer incidents of laryngeal vestibule penetration and lower (better) Penetration‐Aspiration scores (Pelletier and Lawless 2003; Lee et al. 2012), and fewer incidents of aspiration (Pelletier and Lawless 2003). In a study of the effects of enhanced bolus tastes on oropharyngeal swallow in patients treated for head and neck cancer (Pauloski et al. 2013), sour taste significantly shortened PTT across all evaluations. Sour taste administered to the whole mouth during active swallowing appears to be quite robust in augmenting swallow function in healthy control subjects and in improving timing of the pharyngeal swallow in patients with either peripheral or central sensory damage.

Despite this growing body of research examining taste administered to the whole mouth in active swallowing, there has been little study of sensory recognition of taste in the oral cavity alone as it relates to the presence and magnitude of the delay in triggering of the pharyngeal swallow in specific patient types and normal subjects across the age span. Although it has been demonstrated that the pharyngeal swallow can be elicited by stimulation of the structures innervated by the glossopharyngeal nerve, it is not at all clear how sensory stimulation of the oral tongue relates to the pharyngeal swallow. Given that afferent fibers sensitive to taste from CN VII synapse in the NTS, it seems possible that taste stimulation of the oral tongue could activate the swallow CPG. The impact of sour taste on active swallowing has demonstrated changes in the timing of pharyngeal swallow events. However, whole‐mouth stimulation with a sour bolus likely activated both facial and glossopharyngeal sensory fibers.

As the impact of sensory stimulation of the oral tongue alone on pharyngeal swallowing events has not been demonstrated, the purpose of this study was to relate timing of baseline oropharyngeal swallow events with measures of oral sensation by which subjects with impaired swallowing function were able to detect specific tastes of varying intensity. It was hypothesized that there would be a correlation between subjects’ ability to detect sour and sweet tastes on the oral tongue in isolation and timing of the oropharyngeal swallow. The findings could demonstrate a link between swallow motor function and oral sensation, highlighting a potential sensory origin of dysphagia that can be used to develop screening procedures and innovative therapeutic approaches to compensate for the disorder. Moreover, understanding of such relationships will help situate and understand swallowing in the broader parlance of human motor control.

## Methods

### Subjects

Study participants included six subjects referred as outpatients for a videofluorographic (VFG) evaluation of the swallow using the modified barium swallow (MBS) procedure (Logemann 1993) between 17 October and 30 October 2014. Two men and four women participated in the study. The subjects were enrolled consecutively from outpatients referred to Northwestern University's Voice, Speech, and Swallowing Clinic for a swallow evaluation. All outpatients were eligible for the study if able to understand instructions in English and participate in the oral sensory task (described below) within 1 h of completing the videofluorographic swallow evaluation. The subjects ranged in age from 39 to 83 years (mean age of 70 years). Subject 1 was an 83‐year‐old male with a complaint of difficulty swallowing and coughing after eating for the past month. He had a history of a distal esophageal stricture that was dilated 4 months previously. Subject 2 was a 74‐year‐old female with complaint of coughing spells while eating and drinking as well as on saliva occasionally. She had a left frontal/subinsular stroke 16 months prior to the MBS study. Subject 3 was a 39‐year‐old female with no remarkable medical history but complaint of recent difficulty swallowing solid foods, especially meat. Subject 4 was an 82‐year‐old male who had a right hemisphere stroke 3 months prior to the MBS examination used for this study. During an MBS examination 2 weeks after his stroke, he presented with thin liquid aspiration; he was currently receiving outpatient swallow therapy. Subject 5 was a 71‐year‐old female with a complaint of difficulty swallowing and a feeling of food sticking in her throat. She had no remarkable medical history. Finally, subject 6 was a 77‐year‐old female who had been discharged recently from the hospital for breathing difficulty. She had received a gastrostomy tube during her hospitalization which was still in place and being used by patient at time of MBS study. The patient had a history of left true vocal fold paralysis and chronic obstructive pulmonary disease.

### Study protocol

All procedures were approved by the Institutional Review Board for studies involving human subjects at Northwestern University and written consents were obtained from all of the study participants. Each subject was examined once during a single visit and completed the following tasks:

#### MBS procedure with videofluorography (VFG)

The MBS study protocol included swallows of thin liquid barium (Bracco Diagnostics Varibar liquid, Bracco Diagnostics, Inc., Monroe Township, NJ, USA) in several volumes (1, 3, 5, 10 mL) as tolerated, self‐selected volume of thin liquid barium from a cup, and 3 mL barium paste (Bracco Diagnostics Varibar Pudding). Two trials of each bolus size/type were given as tolerated by the subject. The VFG studies were conducted in the lateral plane according to the procedure outlined by Logemann (1993), and recorded at 30 frames per second. Subjects were referred for the swallow examination by their physician and the swallow studies were performed as standard care rather than for research purposes. Bracco Diagnostics describes their barium liquid as having “a pleasing apple flavor” and their barium puddings as having “a pleasing vanilla flavor” for patient compliance. No modifications to the taste of the barium products were made for this study; that is, bolus flavor was not enhanced with sour or sweet taste.

#### Oral sensory testing

Assessment of sensory function associated with taste was done on each subject. Oral sensory testing was performed within 1 h after completion of the MBS evaluation.

##### Taste detection threshold

Subjects were tested to determine taste detection threshold (Fukunaga et al. 2005). Sweet (sucrose) and sour (citric acid) taste stimuli at concentration levels given in Table 1 were prepared in aqueous solution and kept in a glass jar at room temperature. The solutions were all odorless. Sweet and sour taste detection thresholds were tested separately. The order in which the tastants (i.e., sweet or sour) were tested was randomly selected for each subject. The subject was seated comfortably with the back supported. First, each subject rinsed the mouth thoroughly with water and expectorated into an opaque beaker. Then the subject was instructed to stick out the tongue and hold it in place between the upper and the lower incisors. Three droplets of the stimulus solution were placed at the tongue tip by the experimenter with a 1 mL syringe. The subject returned the tongue back to the mouth; then the experimenter asked the subject “What do you taste?” The stimuli were applied in ascending order of concentration level. If the subject indicated that he or she tasted nothing, the next level of concentration was administered. Once the subject successfully identified the tastant, the next level of concentration was given. Once the subject successfully recognized the tastant in three consecutive concentration levels, the task was ended. The lowest concentration level in the series was taken to be the detection threshold.

**Table 1:** Concentration levels of sweet and sour stimuli

Solution no.	Sucrose (mol/L)	Citric acid (mol/L)
1	0.005	0.005
2	0.01	0.01
3	0.03	0.03
4	0.05	0.05
5	0.1	0.1
6	0.3	0.3
7	0.5	0.5
8	1.0	1.0

##### Taste detection of mixtures

The subject next participated in a two‐alternate forced‐choice identification test on a continuum of taste mixtures (Maier and Katz 2013). The nine‐step continuum spanned the sweet and the sour tastes and was a mixture of 0.8 mol/L sucrose and 0.8 mol/L citric acid solutions at the following ratios: 100%/0%, 90%/10%, 75%/25%, 60%/40%, 50%/50%, 40%/60%, 25%/75%, 10%/90%, 0%/100%. The taste mixtures allowed us to assess the effect of more than one tastant simultaneously and thereby build a more complex taste profile by examining the ability to detect one tastant over the other. The choice of 0.8 mol concentration level was chosen because at this concentration subjects can reliably recognize the taste stimulus (Maier and Katz 2013). In addition, high concentrations of citric acid were effective in speeding aspects of the pharyngeal swallow in various types of subjects during active swallowing (Logemann et al. 1995; Cola et al. 2012; Lee et al. 2012; Pauloski et al. 2013). In order to further define the effects of this type of stimulation in isolation, high concentrations also were chosen for this study. Taste stimuli were again presented as three drops on the tongue tip. The subject was asked “Does that taste sweet or sour?” Each of the taste mixtures was presented three times for a total of 27 trials. Subjects were provided with a generous supply of water along with a basin; they were instructed to rinse and expectorate between each trial. The order of presentation of the trials was randomized.

### Data analysis

#### Outcome measures from VFG

Video recordings of the swallow studies were viewed in slow motion and frame‐by‐frame to obtain timing information to compute the swallowing outcome measures as follows:

##### Oral transit time

The time in seconds (s) it takes the bolus to move through the oral cavity, measured from the first backward movement of the bolus until the head of the bolus passes the point where the ramus of the mandible crosses the tongue base.

##### Pharyngeal delay time (PDT)

The time in seconds (s) until the pharyngeal swallow triggers, measured from the time the head of the bolus passes the ramus of the mandible until the onset of laryngeal elevation.

##### Pharyngeal transit time

The time in seconds (s) required for the bolus to move through the pharynx, measured from the time the head of the bolus passes the ramus of the mandible until the tail of the bolus leaves the cricopharyngeal region.

#### Quantification of oral sensation

Subjects’ responses for the “sour” sensation were used to generate the psychometric functions for taste. The psychometric functions depict the response probabilities for each of the nine taste stimuli. The areas of the function are used as a measure of the differential ability to detect the sour and the sweet tastes.

### Statistical analysis

In order to evaluate the relationship between swallow kinematics and oral sensitivity, Pearson's correlation coefficients were computed between outcome measures of VFG and measures of oral sensation (Matlab by MathWorks Inc., Natick, MA, USA). Because the MBS study protocol called for two trials of each bolus size and consistency, there was a potential for 12 observations among the six patients for each VFG outcome measure on each bolus size/consistency combination. In order to avoid inappropriately inflating the degrees of freedom by using repeated measures, the values of the two trials per bolus for each VFG outcome measure were averaged, and the patient's average value was correlated with his or her oral sensation measure.

## Results

**Figure 1:**
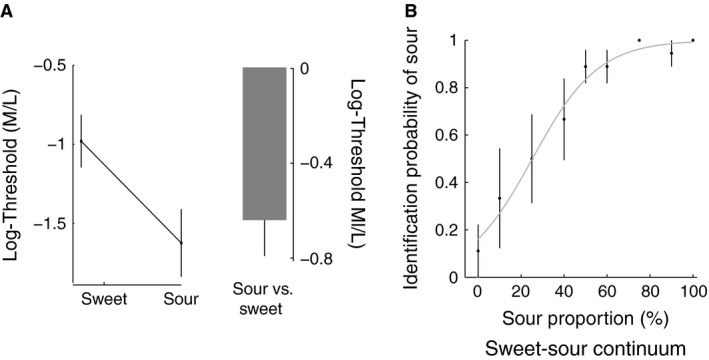
Measures of oral sensation of taste. A. Sweet and sour threshold. The sour threshold is lower than the sweet threshold across the subjects. The right panel shows the difference between the sour and the sweet thresholds. B. Tendency to identify sour sensation in a two‐alternate forced‐choice identification test. The ordinate depicts the probability identifying a stimulus as sour. Stimuli were drawn from a sweet‐sour continuum with mixtures of sweet and sour tastes at varying proportions. Vertical bar represents SE.

Taste detection thresholds were converted to logarithms in order to transform the wide range of concentration values to a more reasonable scale for analysis. The logarithm of a proper fraction is always a negative number, since the log of 1 is zero. The lowest concentration at which sensation is detected provides the sensitivity threshold. One patient was not able to complete the taste detection threshold task because of time limitations; therefore the threshold data were calculated for five subjects. Figure 1A shows sweet and sour thresholds (in logarithms of tastant concentration) averaged across the subjects. For a tastant, the more negative the number, the lower the concentration level, indicating greater sensitivity for recognizing its taste. The mean concentration level for detection of the sour stimuli was −1.6 ± 0.25 (mean ± SE), whereas the mean concentration level for detection of the sweet stimuli was −0.95 ± 0.2 (mean ± SE). There was significantly higher sensitivity, that is, a lower detection threshold, for the sour taste (*t*‐test; *P* < 0.02).

Figure 1B shows results averaged across subjects from the two‐alternate forced‐choice identification test in which subjects had to identify a stimulus drawn from a mixture of sweet‐sour continuum either as sweet or sour. Data were available for all six patients. The abscissa represents the sour proportion of the stimuli presented and the ordinate indicates the probability of identifying the stimuli as sour. A logistic function fitted to the data shows a higher response probability for identifying a stimulus as sour; this result is also consistent with the threshold results obtained. It can also be seen that the identification probability is more variable towards the sweet end of the continuum. For each subject a measure of the tendency to identify a mixture as sour was obtained by averaging across the identification probabilities of the stimuli presented. In all, three sensory parameters were obtained for each subject: detection threshold for the sweet taste; detection threshold for the sour taste; and likelihood of identifying a sweet‐sour mixture as sour, which will be referred to as bias for sour.

A total of 45 swallows during the MBS study were recorded for the six patients. Because the MBS study is tailored to the needs of the individual patient, not every patient in the study was given two trials of each bolus size and consistency. Therefore, there were different numbers of swallows available for analysis depending upon the bolus type. In addition, not all VFG outcome measures could be made on every swallow. The anterior oral cavity was initially out of view on five of the swallows, prohibiting calculation of OTT. For each bolus size and consistency, swallow outcome measures (OTT, PDT, and PTT) from the two trials were averaged for each subject, and the average value was used in subsequent correlational analyses. If a patient had only one trial for a particular bolus size/consistency combination, then the value for the single swallow was used for analysis. The individual data for each bolus as well as the averaged trials for each patient by bolus type and size are included for the reader's inspection in Data S1.

Table 2 summarizes the Pearson correlation coefficients between measures of oral sensation and swallow outcome measures of OTT, PDT, and PTT on 3 mL liquid and 3 mL paste boluses with. Because of the small number of subjects and the likelihood of making a type 1 error with each additional test, the correlation analysis was limited to 3 mL liquid and 3 mL paste because that permitted examination of two bolus consistencies of the same volume. A significant negative correlation was found between PDT on liquid and threshold detection level for the sweet taste, indicating that the lower the subject's detection threshold for sweet, the longer the subject's PDT for liquid.

**Table 2:** Pearson correlation coefficients between perceptual measures of taste and measures of oral transit time (OTT), pharyngeal delay time (PDT), and pharyngeal transit time (PTT) for 3 mL liquid and 3 mL paste boluses

	Consistency	Kinematic parameters		
		OTT	PDT	PTT
Perceptual parameters				
Sweet threshold	3 mL liquid	0.08 (0.90)	−0.94 (0.02)*	0.20 (0.74)
		0.36 (0.55)	−0.49 (0.41)	−0.37 (0.53)
	3 mL paste			
Sour threshold	3 mL liquid	−0.17 (0.79)	−0.77 (0.13)	−0.51 (0.38)
		−0.19 (0.76)	0.26 (0.67)	0.34 (0.58)
	3 mL paste			
Bias for sour	3 mL liquid	0.21 (0.74)	−0.73 (0.16)	0.58 (0.30)
		0.43 (0.40)	−0.83 (0.04)*	−0.77 (0.07)
	3 mL paste			

*P*‐value is in parenthesis. * indicates *P*< .05 level of significance.

**Figure 2:**
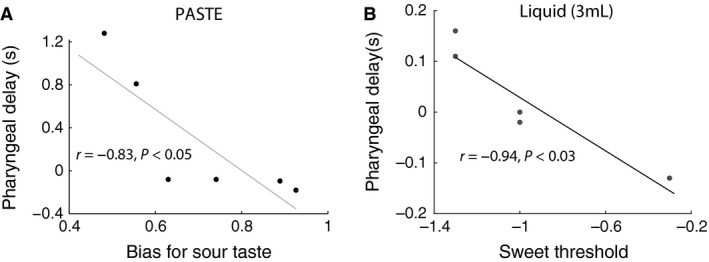
Relationship between oral sensation of taste and pharyngeal swallow events. Greater tendency to identify the sour taste was significantly correlated with shorter pharyngeal delay time for 3 mL paste (A). A lower detection threshold for sweet taste was significantly correlated with longer pharyngeal delay time for 3 mL liquid (B).

A significant negative correlation also was found between PDT for paste and tendency to identify sour taste (Fig. 2). Subjects with greater tendency to identify a mixture as sour tended to have smaller PDTs. In other words, heightened response bias for the sour taste was a strong predictor of shorter pharyngeal delay, that is, better swallow function. Taken together, these results suggest a broad association between taste sensation of the oral tongue and pharyngeal swallow function.

## Discussion

Several measures of oral sensation (sweet and sour detection thresholds, likelihood of identifying a sweet‐sour mixture as sour) were related to swallow kinematics. A significant correlation was found between detection threshold of sweet taste and duration of pharyngeal delay on liquids. In addition, significant correlations were observed between PDT on paste boluses and the ability to detect the sour taste on the oral tongue. Although a reflexive pharyngeal swallow and laryngeal adduction may be activated by stimulation of the internal branch of the superior laryngeal nerve and the pharyngeal branch of the glossopharyngeal nerve (Ludlow et al. 1992; Barkmeier et al. 2000; Kitagawa et al. 2002), the ability to elicit a pharyngeal swallow response via sensory stimulation of the oral tongue has not yet been demonstrated. Given the afferent inputs from the facial nerve via the chorda tympani to the NTS, it is likely that taste sensation from the oral tongue could activate the swallow CPG. Our results suggest a potential link between oral sensation of taste from the oral tongue and pharyngeal swallow motor function.

Although there are variations in taste perception threshold around the perimeter of the tongue, these variations are small; essentially, sweet, sour, bitter, and salty tastes can be perceived in all loci where there are taste receptors (Bartoshuk 1993). Nevertheless, there is a greater concentration of receptors more sensitive to sweet stimuli on the tongue tip and dorsum, whereas sour is better sensed on the palate. In addition, more chorda tympani taste sensory fibers respond to sweet stimuli than to salty, sour, or bitter (Miller 1999b). Given these facts, administration of the taste stimuli to the tongue tip could theoretically bias the results toward greater perception of sweet rather than sour. Indeed, one significant association was observed between detection threshold for sweet taste and duration of pharyngeal swallow delay on liquids. The direction of this relationship was unexpected. Lower detection threshold for the sweet stimulus was significantly correlated with longer PDT for liquid. Studies examining the effect of a sweet taste stimulus on swallow function have yielded contradictory results. In animal studies, a sucrose solution applied to the mucosa of the pharyngolaryngeal region did not elicit any more successive reflexive swallows than did distilled water (Kajii et al. 2002). In humans, use of a sweet taste did not have any differential effect upon magnitude, timing, or duration of surface electromyography of the suprahyoid musculature (Miyaoka et al. 2006), swallow apnea duration (Butler et al. 2004), or transit and clearance durations or residue in the oral cavity, pharynx, or esophagus in healthy control subjects (Alves et al. 2013) or in those with stroke (Alves et al. 2014). On the other hand, a sweet bolus did shorten PTT in healthy controls and treated head and neck cancer patients when compared to an unenhanced barium bolus (Pauloski et al. 2013). The relationship between sweet taste sensed on the oral tongue and timing of pharyngeal swallow events needs further examination.

Our results also demonstrated a greater tendency to detect sour taste in the sweet‐sour mixture, and a significant relationship between that tendency and shorter PDTs on 3 mL paste boluses. It is possible that the high concentration of citric acid in the study test solutions, in addition to stimulating taste receptors in the chorda tympani, was also causing trigeminal stimulation (irritation), as acids in high concentration have irritant as well as taste properties (Prescott et al. 1984). Besides areas of the principal and spinal trigeminal nuclei, sensory neurons of the trigeminal nerve also project to the NTS (Pfaller and Arvidsson 1988; Jean 1991; Marfurt and Rajchert 1991). Therefore, regardless of the mechanism – taste via the chorda tympani or irritation via the lingual branch of the trigeminal nerve – sensory stimulation of the oral tongue may contribute to activation of the swallow CPG and have an impact on timing of the pharyngeal swallow.

In this small study, a number of significant correlations were obtained between measures of oral sensation and swallow kinematics. It is likely that in a study with a larger, more homogeneous sample, these associations could be further clarified. Moreover, a larger study could examine other measures of oral sensation such as temperature and pressure that could correlate with swallow kinematics. This study, for example, did not include variations in temperature. Identification of oral sensations with the greatest effects on swallowing could be a basis for developing sensory‐based therapeutic interventions for dysphagia. Previous studies have shown that the heightening of certain types of oral sensation such as temperature (Lazzara et al. 1986; Bisch et al. 1994; Michou et al. 2012), taste (Logemann et al. 1995; Pelletier and Lawless 2003; Cola et al. 2012; Lee et al. 2012; Pauloski et al. 2013), carbonation (Bülow et al. 2003; Michou et al. 2012; Moritak et al. 2014), and touch (Lazzara et al. 1986; Lim et al. 2009; Regan et al. 2010) is efficacious in reducing PDT or PTT. These studies together with the present findings confirm that impairment of oral sensation can be a significant contributing factor to pharyngeal swallow delay.

The results of this study – identification of the potential link between oral tongue sensation and pharyngeal swallow motor function – have the potential to provide clinicians with a bedside test of oral sensation that can identify which aspects of oral sensory loss or reduction are related to and may be predictive of impairment of the pharyngeal swallow. From the sensory data and their potential relationship with the pharyngeal swallow, therapy techniques, and especially tools based on sensory training can be developed which may be more effective than what is currently used to ameliorate pharyngeal swallowing problems. For example, thickening liquids for patients with pharyngeal swallow delay is one technique currently widely used to address the problem. The financial impact of using thickened liquids may be substantial. In addition to the cost, some investigators have determined that patients dislike and often do not comply with the recommendation for thickened liquids (Colodny 2005; Macqueen et al. 2003; Garcia et al. 2005). Work on patients with dementia and Parkinson's disease with or without dementia revealed the prevalence of patient dislike for thickened liquids (Logemann et al. 2008; Robbins et al. 2008). In addition, thickening liquids can affect the patient's hydration status if they dislike the thickening (Robbins et al. 2008).

The preliminary results of this study suggest other options may exist for therapeutic intervention for delayed pharyngeal swallow. Improving our understanding of the relationship between a patient's oral sensory awareness on selected bolus attributes and disorders of the pharyngeal swallow should enable us to facilitate the patient's sensory input prior to swallow and thus develop new and improved evaluation and treatment procedures for this disorder. If the oral sensory parameters associated with a timely pharyngeal swallow can be determined, this knowledge can be used to help develop treatment protocols to improve swallow function. If there is a relationship between oral sensation to the bolus characteristic of taste, those related parameters can be used as preswallow stimuli in swallowing therapy. For example, patients with demonstrated pharyngeal swallow delay could be given small volumes of a half and half mixture of lemon juice and water prior to eating and throughout the meal. The sour taste could alert the nervous system to an upcoming swallow. Also, there is some evidence that the effects of a sour taste on swallowing function may persist beyond the initial presentation (Kajii et al. 2002; Wahab et al. 2010, 2011).

The results of this study may also be expanded in a larger trial to help identify the concentration of sour taste required for threshold awareness, therefore guiding the proportion of lemon and water needed to elicit a normal pharyngeal response time. This study used stimuli at the sensory threshold level. The study could be expanded to assess the relationship between suprathreshold level stimuli and pharyngeal swallow characteristics. In addition, one or more oral sensory response characteristics may also be useful as a screening tool for identifying those patients who could possibly have a pharyngeal swallow delay and should be followed with a diagnostic imaging study such as the MBS procedure. Future work may also expand the type of sensory testing beyond taste to properties such as bolus volume, viscosity and surface texture (e.g., smooth, firm, crunchy). One or more of these attributes may be related to pharyngeal swallow response including pharyngeal bolus transit or pharyngeal residue.

Recent works from speech motor learning demonstrate a close link between speech perception and production. Speech motor learning appears to alter auditory perception of speech (Nasir and Ostry 2009; Shiller et al. 2009). Based on the preliminary data presented in this study it is likely that a similar link also exists between swallowing and oral sensation and, if so, oral sensory training can be utilized to elicit positive sustainable effects on swallow motor function (Darainy et al. 2013).

This study was exploratory in nature, with the aim to examine a potential link between taste sensations of the oral tongue and swallow kinematics. As such, there are limitations to this study which will be addressed in a larger trial. First, the group of patients studied was undoubtedly small and heterogeneous. Subjects varied in age, medical history, and reason for referral for the clinical swallow evaluation. A homogeneous group of patients could have allowed for stability of covariates such as disease and age. However, as the goal of this study was to examine a possible relationship between oral sensitivity for taste in isolation and measures of timing of pharyngeal swallow events, a range of function could be helpful in detecting such a relationship. Replication of the study with a larger group of homogenous subjects will provide the possibility of detecting stronger associations between oral tongue sensations and swallow kinematics or even different patterns of association depending upon diagnosis. Replication of the study with a larger number of subjects is an important step in the process of establishing the link between oral tongue sensation and pharyngeal swallow function.

Subject factors including age, gender, and genetic variation in taste sensitivity need to be controlled in future studies. There is evidence that ability to detect taste varies with age (Hyde and Feller 1981; Chauhan and Hawrysh 1988; Mojet et al. 2001, 2003; Fukunaga et al. 2005; Heft and Robinson 2010; Imoscopi et al. 2012; Methven et al. 2012), gender (Hyde and Feller 1981; Chauhan and Hawrysh 1988; Mojet et al. 2001, 2003; Heft and Robinson 2010), and genetic variation in taste sensitivity to 6‐n‐propylthiouracil (PROP) (Bartoshuk et al. 1994; Bartoshuk 2000; Tepper et al. 2009; Hayes and Keast 2011; Steele et al. 2012). In general, there is a decline in taste perception with increasing age, while women tend to have greater sensitivity to taste than do men. Genetic differences in the ability to detect PROP have resulted in the classification of two to three groups called “supertasters,” “medium tasters,” and “nontasters” who demonstrate significant differences in the perception of tastes (Bartoshuk et al. 1994; Bartoshuk 2000; Tepper et al. 2009; Hayes and Keast 2011). A test using edible taste strips can be used to identify a subject's “taster” status (Smutzer et al. 2013). In future studies, this test will be included in the protocol to determine each subject's classification. Then, this classification along with age and gender will be used as covariates in the statistical analyses.

The opportunities to examine the relationships among oral and oropharyngeal sensation and pharyngeal swallow motor events are wide‐ranging. In addition to studying the relationships in patient populations, larger studies in healthy adults are needed as well, with consideration of different afferent nerve pathways, including not only the chorda tympani but also the internal branch of the superior laryngeal nerve and the glossopharyngeal nerve. Clearly there are many potential methodologies and protocols to be developed in the pursuit of elucidating the mechanisms of oral sensation and the pharyngeal swallow.

## Conflict of Interest

None declared.

## Supporting information




**Data S1.** All Individual Subject Data and Subject Data Averaged Over Multiple Trials of Bolus Size/Consistency.
