# Supplementary material for: Orosensory contributions to dysphagia: a link between perception of sweet and sour taste and pharyngeal delay time
Source: Physiol Rep. 2016 Jun 14;4(11):e12752. doi: 10.14814/phy2.12752 (PMC4908483; doi:10.14814/phy2.12752)
Supplement: Supplementary file 1 — Data S1. All Individual Subject Data and Subject Data Averaged Over Multiple Trials of Bolus Size/Consistency. [file PHY2-4-e12752-s001.docx]

Supplement

| **Subject data Averaged Over Multiple Trials of Bolus Size/Consistency** | | | | | | | | |  |
| --- | --- | --- | --- | --- | --- | --- | --- | --- | --- |
| **(“.” indicates missing values; see text for details)** | | | | | | | | |  |
| **subj. no.** | **sweet thres-hold** | **sour thres-hold** | **sour bias** | **consistency** | **bolus size** | **oral transit time (s)** | **pharyngeal delay time (s)** | **pharyngeal transit time (s)** |  |
| **1 mL liquid** | |  |  |  |  |  |  |  |  |
| 1 | . | . | 0.8889 | liquid | 1 mL | 0.25 | 1.03 | 1.68 |  |
| 2 | -1.3010 | -2.3010 | 0.6296 | liquid | 1 mL | 0.53 | 0.56 | 1.145 |  |
| 3 | -1.3010 | -1.5229 | 0.4815 | liquid | 1 mL | 0.585 | 0.665 | 1.165 |  |
| 4 | -0.3010 | -1.0000 | 0.9259 | liquid | 1 mL | 0.55 | 0.14 | 1.025 |  |
| 5 | -1.0000 | -1.3010 | 0.5556 | liquid | 1 mL | 0.715 | 0.23 | 0.4 |  |
| 6 | -1.0000 | -2.0000 | 0.7407 | liquid | 1 mL | 2.3 | -0.075 | 0.885 |  |
| **3 ml liquid** | |  |  |  |  |  |  |  |  |
| 2 | -1.3010 | -2.3010 | 0.6296 | liquid | 3 mL | . | 0.16 | 0.93 |  |
| 3 | -1.3010 | -1.5229 | 0.4815 | liquid | 3 mL | 0.36 | 0.11 | 0.7 |  |
| 4 | -0.3010 | -1.0000 | 0.9259 | liquid | 3 mL | 0.44 | -0.13 | 0.865 |  |
| 5 | -1.0000 | -1.3010 | 0.5556 | liquid | 3 mL | 0.81 | -0.02 | 0.81 |  |
| 6 | -1.0000 | -2.0000 | 0.7407 | liquid | 3 mL | 1.21 | 0 | 0.465 |  |
| **5 ml liquid** | |  |  |  |  |  |  |  |  |
| 1 | . | . | 0.8889 | liquid | 5 mL | . | 0.24 | 1.07 |  |
| 2 | -1.3010 | -2.3010 | 0.6296 | liquid | 5 mL | 0.565 | -0.05 | 0.73 |  |
| 3 | -1.3010 | -1.5229 | 0.4815 | liquid | 5 mL | 0.38 | 0 | 0.655 |  |
| 4 | -0.3010 | -1.0000 | 0.9259 | liquid | 5 mL | 0.39 | -0.1 | 0.81 |  |
| 5 | -1.0000 | -1.3010 | 0.5556 | liquid | 5 mL | 0.835 | -0.01 | 0.8 |  |
| **10 ml liquid** | |  |  |  |  |  |  |  |  |
| 1 | . | . | 0.8889 | liquid | 10 mL | 0.545 | -0.03 | 0.625 |  |
| 2 | -1.3010 | -2.3010 | 0.6296 | liquid | 10 mL | . | -0.035 | 0.74 |  |
| 5 | -1.0000 | -1.3010 | 0.5556 | liquid | 10 mL | 0.4 | 0.025 | 0.815 |  |
| **3 mL Paste** | |  |  |  |  |  |  |  |  |
| 1 | . | . | 0.8889 | pudding | 3 mL | 1.06 | -0.095 | 0.565 |  |
| 2 | -1.3010 | -2.3010 | 0.6296 | pudding | 3 mL | 2.05 | -0.08 | 0.565 |  |
| 3 | -1.3010 | -1.5229 | 0.4815 | pudding | 3 mL | 0.425 | 1.28 | 2.025 |  |
| 4 | -0.3010 | -1.0000 | 0.9259 | pudding | 3 mL | 2.62 | -0.18 | 0.755 |  |
| 5 | -1.0000 | -1.3010 | 0.5556 | pudding | 3 mL | 0.61 | 0.81 | 1.475 |  |
| 6 | -1.0000 | -2.0000 | 0.7407 | pudding | 3 mL | 5.28 | -0.08 | 0.945 |  |
|  |  |  |  |  |  |  |  |  |  |
|  |  |  |  |  |  |  |  |  |  |

| **All Individual Subject Data** | | | | | | | | | |
| --- | --- | --- | --- | --- | --- | --- | --- | --- | --- |
| **(“.” indicates missing values; see text for details)** | | | | | | | | |  |
| **subj. no.** | **sweet thres-hold** | **sour thres-hold** | **sour bias** | **swallow trial** | **consistency** | **bolus size** | **oral transit time (s)** | **pharyngeal delay time (s)** | **pharyngeal transit time (s)** |
| 1 | . | . | 0.8889 | 1 | liquid | 1 mL | 0.25 | 1.03 | 1.68 |
| 2 | -1.3010 | -2.3010 | 0.6296 | 1 | liquid | 1 mL | 0.45 | 0.58 | 1.23 |
| 2 | -1.3010 | -2.3010 | 0.6296 | 2 | liquid | 1 mL | 0.61 | 0.54 | 1.06 |
| 3 | -1.3010 | -1.5229 | 0.4815 | 1 | liquid | 1 mL | 0.8 | 0.7 | 1.15 |
| 3 | -1.3010 | -1.5229 | 0.4815 | 2 | liquid | 1 mL | 0.37 | 0.63 | 1.18 |
| 4 | -0.3010 | -1.0000 | 0.9259 | 1 | liquid | 1 mL | 0.75 | -0.19 | 0.71 |
| 4 | -0.3010 | -1.0000 | 0.9259 | 2 | liquid | 1 mL | 0.35 | 0.47 | 1.34 |
| 5 | -1.0000 | -1.3010 | 0.5556 | 1 | liquid | 1 mL | 0.68 | 0.48 | 0.15 |
| 5 | -1.0000 | -1.3010 | 0.5556 | 2 | liquid | 1 mL | 0.75 | -0.02 | 0.65 |
| 6 | -1.0000 | -2.0000 | 0.7407 | 1 | liquid | 1 mL | 3.05 | -0.08 | 0.85 |
| 6 | -1.0000 | -2.0000 | 0.7407 | 2 | liquid | 1 mL | 1.55 | -0.07 | 0.92 |
|  |  |  |  |  |  |  |  |  |  |
| 2 | -1.3010 | -2.3010 | 0.6296 | 1 | liquid | 3 mL | . | 0.16 | 0.93 |
| 3 | -1.3010 | -1.5229 | 0.4815 | 1 | liquid | 3 mL | 0.19 | 0.2 | 0.8 |
| 3 | -1.3010 | -1.5229 | 0.4815 | 2 | liquid | 3 mL | 0.53 | 0.02 | 0.6 |
| 4 | -0.3010 | -1.0000 | 0.9259 | 1 | liquid | 3 mL | 0.38 | -0.11 | 0.89 |
| 4 | -0.3010 | -1.0000 | 0.9259 | 2 | liquid | 3 mL | 0.5 | -0.15 | 0.84 |
| 5 | -1.0000 | -1.3010 | 0.5556 | 1 | liquid | 3 mL | 0.97 | -0.04 | 0.78 |
| 5 | -1.0000 | -1.3010 | 0.5556 | 2 | liquid | 3 mL | 0.65 | 0 | 0.84 |
| 6 | -1.0000 | -2.0000 | 0.7407 | 1 | liquid | 3 mL | 2.42 | 0 | 0.93 |
|  |  |  |  |  |  |  |  |  |  |
| 1 | . | . | 0.8889 | 1 | liquid | 5 mL | . | 0.24 | 1.07 |
| 2 | -1.3010 | -2.3010 | 0.6296 | 1 | liquid | 5 mL | 0.46 | -0.06 | 0.75 |
| 2 | -1.3010 | -2.3010 | 0.6296 | 2 | liquid | 5 mL | 0.67 | -0.04 | 0.71 |
| 3 | -1.3010 | -1.5229 | 0.4815 | 1 | liquid | 5 mL | 0.4 | 0.02 | 0.68 |
| 3 | -1.3010 | -1.5229 | 0.4815 | 2 | liquid | 5 mL | 0.36 | -0.02 | 0.63 |
| 4 | -0.3010 | -1.0000 | 0.9259 | 1 | liquid | 5 mL | 0.39 | -0.1 | 0.81 |
| 5 | -1.0000 | -1.3010 | 0.5556 | 1 | liquid | 5 mL | 0.98 | 0.02 | 0.82 |
| 5 | -1.0000 | -1.3010 | 0.5556 | 2 | liquid | 5 mL | 0.69 | -0.04 | 0.78 |
|  |  |  |  |  |  |  |  |  |  |
| 1 | . | . | 0.8889 | 1 | liquid | 10 mL | 0.88 | 0 | 0.64 |
| 1 | . | . | 0.8889 | 2 | liquid | 10 mL | 0.21 | -0.06 | 0.61 |
| 2 | -1.3010 | -2.3010 | 0.6296 | 1 | liquid | 10 mL | . | 0 | 0.88 |
| 2 | -1.3010 | -2.3010 | 0.6296 | 2 | liquid | 10 mL | . | -0.07 | 0.6 |
| 5 | -1.0000 | -1.3010 | 0.5556 | 1 | liquid | 10 mL | 0.48 | 0.07 | 0.93 |
| 5 | -1.0000 | -1.3010 | 0.5556 | 2 | liquid | 10 mL | 0.32 | -0.02 | 0.7 |
| **subj. no.** | **sweet thres-hold** | **sour thres-hold** | **sour bias** | **swallow trial** | **consistency** | **bolus size** | **oral transit time (s)** | **pharyngeal delay time (s)** | **pharyngeal transit time (s)** |
| 1 | . | . | 0.8889 | 1 | paste | 3 mL | 0.77 | -0.07 | 0.58 |
| 1 | . | . | 0.8889 | 2 | paste | 3 mL | 1.35 | -0.12 | 0.55 |
| 2 | -1.3010 | -2.3010 | 0.6296 | 1 | paste | 3 mL | 2.05 | -0.1 | 0.59 |
| 2 | -1.3010 | -2.3010 | 0.6296 | 2 | paste | 3 mL | . | -0.06 | 0.54 |
| 3 | -1.3010 | -1.5229 | 0.4815 | 1 | paste | 3 mL | 0.32 | 0.86 | 1.61 |
| 3 | -1.3010 | -1.5229 | 0.4815 | 2 | paste | 3 mL | 0.53 | 1.7 | 2.44 |
| 4 | -0.3010 | -1.0000 | 0.9259 | 1 | paste | 3 mL | 1.08 | -0.2 | 0.74 |
| 4 | -0.3010 | -1.0000 | 0.9259 | 2 | paste | 3 mL | 4.16 | -0.16 | 0.77 |
| 5 | -1.0000 | -1.3010 | 0.5556 | 1 | paste | 3 mL | 0.6 | 0.5 | 1.15 |
| 5 | -1.0000 | -1.3010 | 0.5556 | 2 | paste | 3 mL | 0.62 | 1.12 | 1.8 |
| 6 | -1.0000 | -2.0000 | 0.7407 | 1 | paste | 3 mL | 3.52 | -0.1 | 0.9 |
| 6 | -1.0000 | -2.0000 | 0.7407 | 2 | paste | 3 mL | 7.04 | -0.06 | 0.99 |
